# E-Cigarette Advocates on Twitter: Content Analysis of Vaping-Related Tweets

**DOI:** 10.2196/17543

**Published:** 2020-10-14

**Authors:** Kahlia McCausland, Bruce Maycock, Tama Leaver, Katharina Wolf, Becky Freeman, Jonine Jancey

**Affiliations:** 1 Collaboration for Evidence, Research and Impact in Public Health School of Public Health Curtin University Bentley Australia; 2 College of Medicine and Health University of Exeter Devon United Kingdom; 3 School of Media, Creative Arts and Social Inquiry Curtin University Bentley Australia; 4 School of Marketing Curtin University Bentley Australia; 5 School of Public Health University of Sydney Sydney Australia

**Keywords:** electronic nicotine delivery systems, electronic cigarettes, e-cigarette, infodemiology, infoveillance, vaping, Twitter, social media, public health, content analysis

## Abstract

**Background:**

As the majority of Twitter content is publicly available, the platform has become a rich data source for public health surveillance, providing insights into emergent phenomena, such as vaping. Although there is a growing body of literature that has examined the content of vaping-related tweets, less is known about the people who generate and disseminate these messages and the role of e-cigarette advocates in the promotion of these devices.

**Objective:**

This study aimed to identify key conversation trends and patterns over time, and discern the core voices, message frames, and sentiment surrounding e-cigarette discussions on Twitter.

**Methods:**

A random sample of data were collected from Australian Twitter users who referenced at least one of 15 identified e-cigarette related keywords during 2012, 2014, 2016, or 2018. Data collection was facilitated by TrISMA (Tracking Infrastructure for Social Media Analysis) and analyzed by content analysis.

**Results:**

A sample of 4432 vaping-related tweets posted and retweeted by Australian users was analyzed. Positive sentiment (3754/4432, 84.70%) dominated the discourse surrounding e-cigarettes, and vape retailers and manufacturers (1161/4432, 26.20%), the general public (1079/4432, 24.35%), and e-cigarette advocates (1038/4432, 23.42%) were the most prominent posters. Several tactics were used by e-cigarette advocates to communicate their beliefs, including attempts to frame e-cigarettes as safer than traditional cigarettes, imply that federal government agencies lack sufficient competence or evidence for the policies they endorse about vaping, and denounce as propaganda “gateway” claims of youth progressing from e-cigarettes to combustible tobacco. Some of the most common themes presented in tweets were advertising or promoting e-cigarette products (2040/4432, 46.03%), promoting e-cigarette use or intent to use (970/4432, 21.89%), and discussing the potential of e-cigarettes to be used as a smoking cessation aid or tobacco alternative (716/4432, 16.16%), as well as the perceived health and safety benefits and consequences of e-cigarette use (681/4432, 15.37%).

**Conclusions:**

Australian Twitter content does not reflect the country’s current regulatory approach to e-cigarettes. Rather, the conversation on Twitter generally encourages e-cigarette use, promotes vaping as a socially acceptable practice, discredits scientific evidence of health risks, and rallies around the idea that e-cigarettes should largely be outside the bounds of health policy. The one-sided nature of the discussion is concerning, as is the lack of disclosure and transparency, especially among vaping enthusiasts who dominate the majority of e-cigarette discussions on Twitter, where it is unclear if comments are endorsed, sanctioned, or even supported by the industry.

## Introduction

The global e-cigarette market was worth US $11.26 billion in 2018 [1] and is predicted to eclipse tobacco sales by 2023 [2]. Facilitating this growth is the increasing trend toward online retailing and social media consumption [3]. Social media has emerged as a popular forum for e-cigarette users (vapers) and prospective users to learn about and share their experiences with nicotine and vaping devices, for businesses to promote their products, and for e-cigarette advocates to debate regulatory regimes [4,5]. Digital media, including social media and social networking platforms, are increasingly preferred sources for health information and dissemination [6]. However, users may be inadvertently exposed to misinformation, disinformation, and unregulated advertising [7,8].

With its 330 million users [9], real-time content updates, and rapid information dissemination, Twitter contributes to e-cigarette marketing and information sharing [10]. As the majority of Twitter content is publicly available, the platform has become a rich data source for public health surveillance providing insights into emergent phenomena, such as vaping [11]. Recent investigations have shown that Twitter users are overwhelmingly exposed to positive messages about vaping, most notably marketing and promotion, and that public health messaging is particularly absent from communications [4]. Although there is a growing body of literature that has examined the content of vaping-related tweets [4,12], less is known about the people who generate and disseminate these messages, and the role of e-cigarette advocates in this promotion.

In Australia, the context of this study, the legal status of e-cigarettes is determined by existing and overlapping laws relating to poisons, therapeutic and consumer goods, and tobacco control [13]. Liquid nicotine is classified as a “Schedule 7-Dangerous Poison” under the Federal Poisons Standard [14], and, as such, the manufacture, sale, or supply of e-cigarettes containing nicotine without lawful authority (ie, prescription from a medical doctor) [15] is prohibited in all Australian states and territories [16]. However, nicotine-containing e-cigarettes are imported into Australia as there is no way to determine whether an e-cigarette contains nicotine without a laboratory analysis, which has implications for law enforcement [16,17]. E-cigarettes that do not contain nicotine can be sold in some Australian jurisdictions, provided manufacturers do not make therapeutic claims.

As of January 2019, there were approximately 2.56 million active monthly Australian Twitter users (64% male), which equates to approximately 12% of Australians over 13 years of age [18]. Given the popularity of Twitter [18], the ease of which information disseminates among its users, and the power of Twitter to traffic users to external webpages [19], insights into how the platform is used (and by whom) to promote and discuss e-cigarettes are warranted. This study aimed to identify key conversation trends and patterns over time and discern the core voices, message frames, and sentiment surrounding e-cigarette discussions in an Australian context. Investigating these public conversations can contribute to understanding trends in knowledge, attitudes, and behaviors; identify marketing strategies; inform public health and public policy; and pave the way for interventions delivered via social media [20-23].

## Methods

### Data Collection

Twitter data were collected via TrISMA (Tracking Infrastructure for Social Media Analysis) [24], a contemporary technical and organizational infrastructure for the tracking of public communication by Australian users of social media. Central to the TrISMA Twitter infrastructure is the Australian Twitter Collection, which continuously gathers tweets from identified Australian accounts (ie, accounts set to an Australian location, geolocation, or time zone, or accounts with a description field referring to an Australian location or containing Australia-specific terms) and stores them in a database available to accredited TrISMA researchers. The TrISMA Twitter Collection is hosted on a cloud-based Google BigQuery database and is accessed through the data visualization tool Tableau. The Australian Twitter Collection filters for known signs of bots, such as accounts with numeric strings in the title, accounts with zero followers, and brand new accounts tweeting or retweeting identical content.

A list of popular e-cigarette–related terms was developed based on peer-reviewed literature [25-30], trending Twitter hashtags, and frequently co-occurring hashtags (ie, hashtags that appeared in the same caption as the root term), which resulted in the following 15 keywords: *cloudchasing, ecig* (includes ecigarette/s), *e-cig* (includes e-cigarette/s), *electroniccig* (includes electroniccigarette/s), *electronic cigarette* (includes electronic cigarettes), *eliquid*, *e-liquid*, *e-juice*, *vape* (includes vaper and vapes), *vaping*, *vapecommunity*, *vapefam*, *vapelife*, *vapenation*, and *vapeporn*. E-cigarette product names were omitted from the search strategy so as not to bias the results to specific brands [22]. A preliminary search revealed there was minimal Twitter activity using these keywords before 2012. Two yearly sampling intervals starting from 2012 to 2018 were therefore chosen to maximize the period of time covered while still being able to see the emergence and decline of trends in the collected data.

Data (tweets), along with metadata information (ie, user name and user follower count) were collected from public Australian Twitter users when a tweet included at least one of the identified keywords from each respective year. Data were downloaded in the form of CSV (comma separated value) files for each keyword and respective year. Social media users tend to include multiple hashtags within their posts, which resulted in duplicate tweets being collected. Duplicate tweets within keyword corpora for each year and across keyword corpora from the co-use of hashtags were removed, resulting in the inclusion of only unique tweets [31]. Data were assigned a number in ascending order and 100 tweets from each keyword corpus for each year were randomly selected for analysis, using an online random sequence generator [32]. Selected data were checked by one researcher (KM) to determine eligibility (ie, written in English and relevant to e-cigarettes). If any of the originally selected 100 tweets did not fit the inclusion criteria, further sampling occurred until 100 eligible tweets were reached. If a keyword corpus had less than 100 tweets, all eligible tweets were included. Retweets (tweets reposted by users) were included in this study, which facilitated the understanding of what information was being circulated by Australian users, even if it originated in another country.

### Ethical Considerations

A particularly salient concern among researchers is whether social media data should be considered public or private data [33]. Twitter is a social networking service in which users broadcast their opinions and commonly use a hashtag to associate their thoughts on a subject with users on the same subject, and therefore, these data are generally referred to as “public data” [33]. For ethical, privacy, and technical reasons, TrISMA does not collect tweets from private accounts or direct messages; therefore, all data collected in this study were publicly available. This study was approved by the Curtin University Human Research Ethics Committee (approval number: HRE2017-0144).

### Developing the Coding Frame

A concept-driven approach (inductive) [34] informed by extant studies [22,23,35-42] was utilized to develop a triaxial coding framework to capture the account users, and the sentiment and theme of the tweets they posted. The coding frame was tested on a random sample of 100 tweets, whereby each tweet was read and assigned codes based upon the concepts presented in the descriptive text, hashtags, and any accompanying images [43]. One researcher (KM) undertook this process in NVivo (v11; QSR International), iteratively revising the coding framework to further refine predefined codes, merge others to create broader codes encompassing several related concepts, and identify new codes arising from the data using a data-driven approach (deductive) [34], which served as a revalidation of earlier coded material [44].

### Coding and Analysis

The modified coding framework was transferred to IBM SPSS Statistics (v22; IBM Corp) and applied to the data by the same researcher. The coding descriptor *user category* characterizes the sender of the tweet and typically involved a detailed inspection of the associated Twitter profile, including the profile picture, bio description, follower-to-following ratio, and tweet history (ie, the content of tweets, number of daily tweets, and ratio of original tweets to retweets) to determine who the user was (Multimedia Appendix 1) [39]. Although data were unique, the poster’s of the data were not necessarily so and could be counted multiple times if their data were collected and selected for analysis. The coding descriptor s*entiment* reflects the stance expressed in the tweet toward e-cigarettes and related products or its users, whether positive, negative, or neutral (Multimedia Appendix 2). The coding descriptor *theme* reflects the theme of the actual content conveyed in the tweet (Multimedia Appendix 3). The text of each tweet and/or the Twitter user handle were explored via Twitter’s search function to examine the profile of the user and any comments attached to the tweet to assist with understanding its context. URLs embedded within tweets were followed. If the URL was active, it was recorded as linking to either social media (eg, Instagram, Facebook, and YouTube) or a website (eg, retail, news, and blog). Each code within the coding framework was a variable in SPSS that functioned as a stand-alone item and was evaluated as either 1 for *present* or 2 for *absent*. *User category* and *sentiment* were mutually exclusive categories (ie, only one selection could be made per category), while the *theme* of the tweet and links to social media and websites were not. The chi-square test (or Fisher exact test if applicable) was used to examine the variation in the content of tweets between years.

## Results

### Sample of Posts

In total, 4432 tweets were analyzed. There were 570 (12.86%) tweets in 2012, 1196 (26.99%) in 2014, 1377 (31.07%) in 2016, and 1289 (29.08%) in 2018.

### Retweets

Of the sample, 25.86% (1146/4432) were retweets, and of these, 79.23% (908/1146) were categorized as having a positive sentiment toward e-cigarettes. Posts by vape retailers or manufacturers (254/1146, 22.16%), e-cigarette advocates (248/1146, 21.64%), and the general public (219/1146, 19.11%) were most often retweeted. The content of the most frequently retweeted posts reflected advertising or promotion of vaping-related paraphernalia, groups, brands, retailers, or manufacturers (374/1146, 32.64%); posts mentioning an e-cigarette brand (248/1146, 21.64%); and posts discussing regulation or policy (246/1146, 21.47%) and the health and safety of e-cigarettes (204/1146, 17.80%).

Reporting of the following results includes both original tweets and retweets unless otherwise specified.

### Sentiment

The vast majority of tweets (3754/4432, 84.70%) reflected positive perceptions toward e-cigarettes and related products or its users. Positive sentiment, however, decreased over time as negative sentiment increased (Table 1).

**Table 1:** Sentiment of data.

Sentiment	Year				Total (N=4432), n (%)
	2012 (N=570), n (%)	2014 (N=1196), n (%)	2016 (N=1377), n (%)	2018 (N=1289), n (%)	
Positive	515 (90.35)	1041 (87.04)	1197 (86.93)	1001 (77.66)	3754 (84.70)
Neutral	36 (6.32)	69 (5.77)	96 (6.97)	125 (9.70)	326 (7.36)
Negative	19 (3.33)	86 (7.19)	84 (6.10)	163 (12.65)	352 (7.94)

### User Category

Vape retailers and manufacturers (1161/4432, 26.20%), the general public (1079/4432, 24.35%), and e-cigarette advocates (1038/4432, 23.42%) posted 73.96% (3278/4432) of the data analyzed (Table 2). The number of tweets posted by vape retailers and manufacturers peaked in 2014 and gradually declined in subsequent years. Similarly, tweets posted by e-cigarette advocates peaked, however, later in 2016 and declined in 2018. The number of tweets posted by news and media sources and public health professionals, researchers, and academics gradually increased over time. Tweets posted by suspicious (suspected “bot”) accounts progressively declined since 2012.

**Table 2:** Twitter user category.

User category	Year				Total (N=4432), n (%)
	2012 (N=570), n (%)	2014 (N=1196), n (%)	2016 (N=1377), n (%)	2018 (N=1289), n (%)	
Vape retailer or manufacturer	147 (25.79)	451 (37.71)	310 (22.51)	253 (19.63)	1161 (26.20)
General public	164 (28.77)	303 (25.33)	286 (20.77)	326 (25.29)	1079 (24.35)
E-cigarette advocate	89 (15.61)	235 (19.65)	439 (31.88)	275 (21.33)	1038 (23.42)
News or media source	1 (0.18)	22 (1.84)	48 (3.49)	147 (11.40)	218 (4.92)
Suspected bot	104 (18.25)	54 (4.54)	46 (3.34)	3 (0.23)	207 (4.67)
Other	36 (6.32)	58 (4.85)	73 (5.30)	34 (2.64)	201 (4.54)
Public health professional, researcher, or academic	2 (0.35)	11 (0.92)	35 (2.54)	127 (9.85)	175 (3.95)
Account not active or user suspended	13 (2.28)	46 (3.85)	73 (5.30)	24 (1.86)	156 (3.52)
Consumer advocacy group	13 (2.28)	1 (0.83)	33 (2.40)	50 (3.88)	97 (2.19)
Health or scientific group	0 (0)	6 (0.50)	22 (1.60)	34 (2.64)	62 (1.40)
Medical doctor, nurse, or group	1 (0.18)	7 (0.59)	6 (0.44)	8 (0.62)	22 (0.50)
Government or politician	0 (0)	2 (0.17)	6 (0.44)	8 (0.62)	16 (0.36)

### Sentiment by User Category

Tweets by the general public (845/1079, 78.31%), suspected bot accounts (185/207, 89.4%), e-cigarette advocates (1007/1038, 97.01%), consumer advocacy groups (95/97, 98%), and vape retailers and manufacturers (1158/1161, 99.74%) were predominantly positive (Table 3). Tweets posted by health and scientific groups (32/62, 52%) and medical doctors and nurses (12/22, 54%) were mostly negative, which contrasts with the proportion of positive tweets posted by other members of the public health community (ie, public health professionals, researchers, and academics [106/175, 60.6%]). Tweets by news and media accounts were mostly neutral (97/218, 44.5%).

**Table 3:** Twitter user category and sentiment of data.

User category	Sentiment			Total, n (%)
	Positive, n (%)	Neutral, n (%)	Negative, n (%)	
Vape retailer or manufacturer	1158 (99.74)	0 (0)	3 (0.26)	1161 (26.20)
Consumer advocacy group	95 (97.94)	1 (1.03)	1 (1.03)	97 (2.19)
E-cigarette advocate	1007 (97.01)	23 (2.22)	8 (0.77)	1038 (23.42)
Suspected bot	185 (89.37)	13 (6.28)	9 (4.35)	207 (4.67)
General public	845 (78.31)	115 (10.66)	119 (11.03)	1079 (24.35)
Other	150 (74.63)	27 (13.43)	24 (11.94)	201 (4.54)
Public health professional, researcher, or academic	106 (60.57)	18 (10.29)	51 (29.14)	175 (3.95)
Government or politician	9 (56.25)	1 (6.25)	6 (37.50)	16 (0.36)
Health or scientific group	19 (30.65)	11 (17.74)	32 (51.61)	62 (1.40)
News or media source	48 (22.02)	97 (44.50)	73 (33.49)	218 (4.92)
Medical doctor, nurse, or group	3 (13.64)	7 (31.82)	12 (54.55)	22 (0.50)
Account not active or user suspended	129 (82.69)	13 (8.33)	14 (8.97)	156 (3.52)
Total	3754 (84.70)	326 (7.36)	352 (7.94)	4432 (100)

### Themes Reflected in the Data

The following narrative reflects on some of the most prevalent themes found in the data. Refer to Multimedia Appendix 4 for all themes.

#### Advertising or Promotion

**Figure 1:**
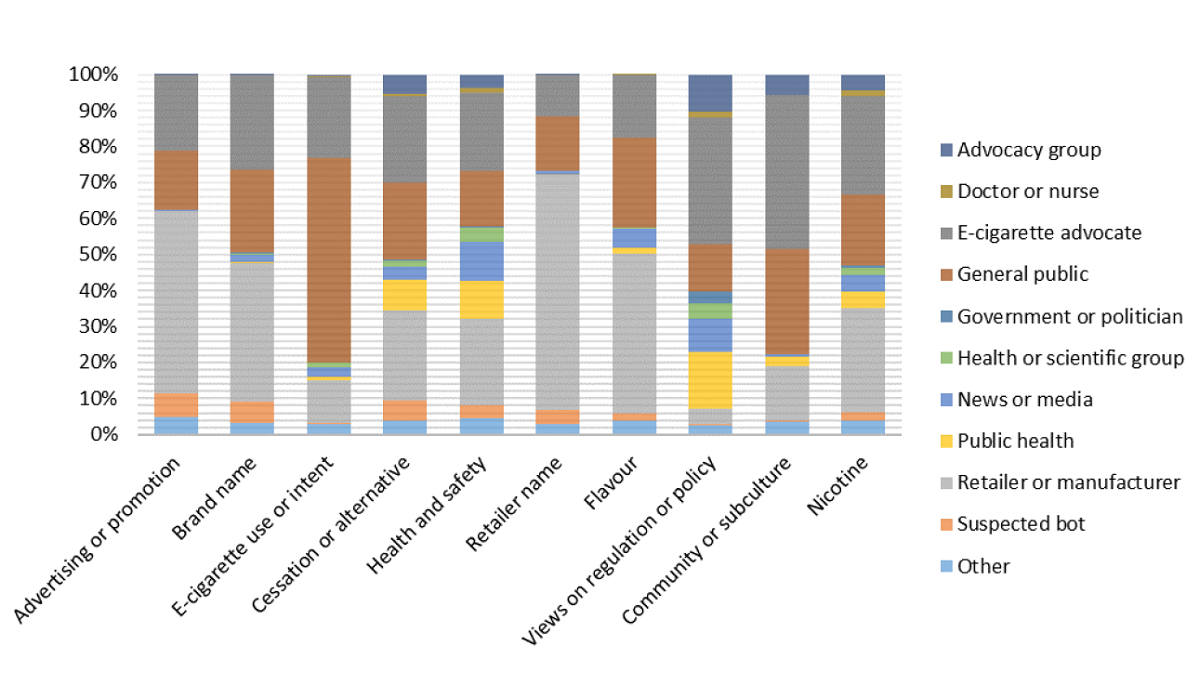
User category contribution in the 10 most prevalent themes.

Almost half (2040/4432, 46.03%) of all data were classified as advertising or promotion. The number of advertising and promotional tweets collected peaked in 2014 and displayed a downward trend in subsequent years (Table 4). These tweets promoted vaping-related paraphernalia, groups, brands, events, and retailers and manufacturers. Strategies used to further promote vape products included providing coupons, discount offers, multibuys, and giveaways. These strategies were collectively coded as price promotions and were present in 19.46% (397/2040) of tweets categorized as advertising or promotion. In 2016, the number of these tweets collected doubled compared with the number collected in other years. E-cigarette retailers and manufacturers (990/2040, 48.53%) and e-cigarette advocates (412/2040, 20.20%) posted the largest proportion of advertising and promotional tweets (Figure 1). Tweets by e-cigarette retailers and manufacturers commonly advertised vaping paraphernalia to purchase as follows:

Have you seen the NS Pen by @VandyVape? Slim and elegant design, and good battery capacity for its size... A great starter kit AVAILABLE in store 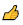 and online! #VandyVape #VapePen #eCig #VapeKit #Vaping #VapeLife #Soulblu

On the other hand, the general public and e-cigarette advocates were inclined to promote and publicize products they were currently using or testing as follows:

Shout out to @VapoureyesNZ you guys always look after me with my regular order of #alpinecloudco #Kosciuszko & your #heisenberg (which honestly is the best I've tried) #loyalcustomer dhl delivery takes 3days & boom my order is here!! #vapefam #vapergirl #vapoureyesnz THANKYOU 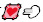

**Table 4:** The 10 most prevalent themes.

Tweet content		Year				Total (N=4432), n (%)
		2012 (N=570), n (%)	2014 (N=1196), n (%)	2016 (N=1377), n (%)	2018 (N=1289), n (%)	
**Advertising or promotion**		268 (47.02)	685 (57.27)	633 (45.97)	436 (33.82)	2040 (46.03)
	Price promotion	77 (28.73)	80 (11.68)	152 (24.01)	88 (20.18)	397 (19.46)
Brand name		124 (21.75)	302 (25.25)	448 (32.53)	364 (28.24)	1238 (27.93)
E-cigarette use or intent		76 (13.33)	254 (21.24)	358 (26.00)	282 (21.88)	970 (21.89)
**Cessation or alternative**		105 (18.42)	182 (15.23)	136 (9.88)	293 (22.75)	716 (16.16)
	Positive	100 (95.24)	176 (96.70)	130 (95.59)	274 (93.52)	680 (94.97)
	Negative	1 (0.95)	4 (2.20)	2 (1.45)	13 (4.44)	20 (2.79)
	Neutral	4 (3.81)	2 (1.14)	4 (2.94)	6 (2.05)	16 (2.24)
**Health and safety**		67 (11.75)	161 (13.46)	139 (10.09)	314 (24.38)	681 (15.37)
	Positive	51 (76.12)	114 (70.81)	91 (65.47)	198 (63.06)	454 (66.66)
	Negative	10 (14.93)	36 (22.36)	36 (25.90)	101 (32.17)	183 (26.87)
	Neutral	6 (8.96)	11 (6.83)	12 (8.63)	15 (4.77)	44 (6.46)
Retailer name		78 (13.68)	234 (19.57)	136 (9.88)	201 (15.61)	649 (14.64)
Flavor		39 (6.84)	145 (12.12)	139 (10.09)	184 (14.29)	507 (11.44)
**Views on regulation or policy**		6 (1.05)	45 (3.76)	64 (4.65)	192 (14.91)	307 (6.97)
	Liberal	3 (50.00)	36 (80.00)	58 (90.63)	151 (78.65)	248 (80.78)
	Cautious	3 (50.00)	6 (13.33)	5 (7.81)	40 (20.83)	54 (17.60)
	Neutral	0 (0)	3 (6.66)	1 (1.56)	1 (0.52)	5 (1.63)
Community or subculture		18 (3.16)	48 (4.01)	84 (6.10)	155 (12.03)	305 (6.88)
Nicotine		19 (3.33)	42 (3.51)	89 (6.46)	143 (11.10)	293 (6.61)

#### A Smoking Cessation Aid or Tobacco Alternative

Overall, 16.16% (716/4432) of tweets discussed the potential of e-cigarettes to be used for tobacco smoking cessation or used as a tobacco alternative. The vast majority of tweets in this category maintained that e-cigarettes could be used to help tobacco smokers quit or reduce their tobacco consumption (680/716, 95.0%), and were most prevalent in 2018 (Table 4). E-cigarette retailers and manufacturers (176/680, 25.9%), e-cigarette advocates (169/680, 24.9%), and the general public (139/680, 20.4%) contributed the largest proportion of tweets supporting the use of e-cigarettes as a smoking cessation product. For example, one retailer posted the following statement:

Thousands of people loosing [sic] their lives because of #Smoking annually. Why don't you #Vape instead of #Smoking which is much safer, in fact it is not at all harmful. Make a move now! #VapeOn #SteamLite

#### Health and Safety

Overall, 15.37% (681/4432) of tweets discussed the perceived health and safety benefits (eg, increased physical stamina and financial wellbeing) and consequences (eg, device malfunction and exacerbation of respiratory diseases) of e-cigarette use. The majority (454/681, 66.7%) of these tweets stated the benefits of using e-cigarettes, peaking in 2018. Similarly, the number of negative health and safety tweets increased over time (Table 4). Tweets considering the positive health and safety aspects of e-cigarettes were dominated by vape retailers and manufacturers (158/454, 34.8%) and e-cigarette advocates (120/454, 26.4%). One post was as follows:

I've been smoke free for almost 5 years now, and have had huge improvements in my health, BECAUSE of switching to vaping, that makes me a criminal in Aus [Australia]. I'll take vaping any day over toxic pharma garbage like pills and gums. Inhaling air is potentially harmful, so is ignorance.

On the other hand, those expressing negative views were news and media sources (52/183, 28.4%), the general public (29/183, 15.8%), and public health professionals, researchers, and academics (27/183, 14.8%). One post was as follows:

As vaping products and their promotion become more prevalent, health professionals are warning that e-cigarettes are not as safe as many people believe.

#### Views on Regulation and Policy

Overall, 6.93% (307/4432) of tweets discussed e-cigarette regulation or policy (Table 4). The majority of the data expressed positive sentiment toward liberal e-cigarette regulation (248/307, 80.8%), and these posts were dominated by e-cigarette advocates (105/248, 42.3%) and public health professionals, researchers, and academics (40/248, 16.1%). One post was as follows:

Long time supporter and campaigner for #vaping I campaigned and worked hard to prevent further restrictions on #vapes Sadly couldn't convince the 3 major parties. Abbreviated policy here https://www.reasonvic.org.au/policy/ #votereason

Consistent with those supporting liberal regulation or legalization, e-cigarette advocates most often provided commentary that challenged the implementation of restrictive e-cigarette policies, provided testimonies as to why e-cigarette regulation should be relaxed, and challenged other Twitter users expressing antivaping views (123/263, 46.8%). One post was as follows:

Anti- #vaping advocates often compare the lack of absolute safety of #ecigs with accepting the precautionary principle as being applicable. Of course this is facile and silly. They ignore the risk of causing harm by blocking innovation by doing nothing.

### Links to Websites and Social Media Platforms

Overall, 44.29% (1963/4432) of posts included at least one embedded URL to an external website. Tweets most frequently included URLs that linked to news (536/1963, 27.31%) and retail websites (415/1963, 21.14%), blogs (326/1963, 16.61%), and scholarly articles and reports (79/1963, 4.02%). Almost half (530/1146, 46.25%) of retweets contained at least one URL that linked to an external website, and the most common were news (169/530, 31.9%) and retail (79/530, 14.9%) websites.

Overall, 18.55% (822/4432) of tweets linked to another social media platform, also known as cross-platform posting. Posts most commonly linked to Instagram (550/822, 66.9%) and Facebook (120/822, 14.6%). Additionally, 22.69% (460/2027) of tweets that were categorized as advertising or promotion were linked to Instagram.

## Discussion

### Principal Findings

This study analyzed 4432 vaping-related tweets from 2012, 2014, 2016, and 2018, posted and retweeted by Australian users. Analysis of the data indicated that positive sentiment continues to dominate the e-cigarette discourse on Twitter, and the ongoing polarized debate among the public health community is not reflected. Largely, a one-sided perspective is being presented by vape retailers and manufacturers, e-cigarette advocates, the general public, and select public health professionals, researchers, and academics.

Twitter users with vested interests in e-cigarettes (ie, retailers and manufacturers), e-cigarette advocates, and the general public were found to tweet a very high proportion of positive tweets (>70%). News and health-related accounts provided messages that were the least positive and/or neutral; however, these tweets comprised a small proportion of the total sample. Our findings concur with recent studies [4,45]. However, we found that some vocal provaping public health professionals, researchers, and academics are skewing the conversation, which is not the view of the wider Australian and international public health communities [46].

E-cigarette advocates, along with a small number of vocal public health professionals, researchers, and academics, were predominately positive in their discussions and were found to challenge other Twitter users who expressed antivaping views or were deemed to be “misrepresenting the facts” concerning e-cigarettes. Some Australian public health academics, who do not support the use of e-cigarettes until they are proven to be a safe and efficacious smoking cessation aid, have documented their relentless struggles with provaping advocates on Twitter [47,48], with one stating that the collective abuse received from other interest groups, such as smokers’ rights advocates, antivaccinationists, and climate change denialists, pales into insignificance compared with the volume of abuse received from vaping advocates. Several tactics were used by e-cigarette advocates to communicate their beliefs, including attempts to frame e-cigarettes as safer than tobacco cigarettes, imply that federal government agencies lack sufficient competence or evidence for the policies they endorse about vaping, and denounce as propaganda “gateway” claims of youth progressing from e-cigarettes to tobacco cigarettes. Australian e-cigarette advocates were also found to use a range of tropes to justify their support for vaping, which have been identified in international research [49], including encouraging an “us versus them” mentality, attacking those opposed to e-cigarettes, relying on personal anecdotal evidence, minimalizing side effects, normalizing use, and emphasizing the benefits of e-cigarettes. These tactics may impact the proportion of the public health community and other Twitter users who are willing to express contradictory views [50], thereby skewing the commentary and possibly shaping the views and risk perceptions of vulnerable populations such as youth [51]. This notion is supported by our findings, with only 7.94% (352/4432) of tweets categorized as negative and 7.36% (326/4432) as neutral.

Groups who are usually viewed as health experts or opinion leaders, such as medical doctors and nurses, reputable scientific organizations, and government organizations and politicians, collectively posted only 2.26% (100/4432) of tweets analyzed in this study. A great deal of health information is now distributed and sourced online, which has resulted in less of a reliance upon these traditional knowledge brokers in offline settings [52]. In the online environment, “the multiplicity of sources involved in information dissemination, their possible anonymity, the absence of standards for information quality, the ease in manipulating and altering content, the lack of clarity of the context, and the presence of many potential targets of credibility evaluation (ie, the content, the source, and the medium)” [52] make the assessment of information an often complex task. As a result, individuals are now burdened with the responsibility of information evaluation that was once the responsibility of professional gatekeepers [53]. The health literacy levels of the Australian population are generally low [54,55], and investigating methods to assist internet users in assessing the credibility of online information is therefore particularly important, as well as the dissemination of evidence-based information by respected experts and opinion leaders.

Our results support previous vaping-related Twitter investigations reporting that the Twitter landscape is dominated by tweets from industry and commercial users championing e-cigarettes as a healthier tobacco alternative and as a successful cessation aid [11,23,41]. These views are contrary to Australia’s regulatory approach to e-cigarettes, which aims to safeguard public health and control the drivers of negative e-cigarette use (ie, use among youth and nonsmokers and unfettered marketing) [56]. Australia is a signatory to the World Health Organization Framework Convention on Tobacco Control, which is designed to protect public health policies from commercial and other vested interests [57]. Until there is adequate evidence that e-cigarettes are safe and an efficacious smoking cessation product, they should not be promoted as such.

A substantial proportion of tweets used sales techniques, such as price promotions, which have historically been successfully employed by the tobacco industry, to influence cigarette uptake and consumption [58]. These findings have implications for the marketing of e-cigarettes on other social media platforms, in particular Instagram, owing to the level of cross-platform interaction found in this investigation, which is worth further examination. Given the substantial youth presence on social media, the marketing of e-cigarettes on these platforms may entice nonsmokers and youth, in particular, to experiment with and initiate vaping [59]. Data from the most recent National Drug Strategy Household Survey [60] reports 11.3% of Australians aged over 14 years have ever used and 2.5% currently use e-cigarettes, with increases of 2.5% and 1.3%, respectively, since 2016. These increases occurred in both smokers and nonsmokers and contrast with Australian combustible smoking rates, which have continued to decline over the last 30 years. The most frequent reason for using e-cigarettes reported by people over 14 years was “out of curiosity” (54.2%). Others (22.8%) cited using e-cigarettes because they perceived them to be less harmful than tobacco cigarettes (19.2% in 2016), and 10.1% believed vaping to be more socially acceptable than tobacco smoking (6.0% in 2016). Further, 26.9% of respondents reported that they obtained their e-cigarette products online (Australian retailer 12.5%, overseas retailer 11.1%, unknown origin 3.3%), a trend that should be closely monitored [61].

### Implications for Public Health

The practice of public health relies on evidence and clear communication between practitioners and the communities they serve [62], and in the absence of balanced evidence-based dialogue, personal opinion and marketing of e-cigarettes dominate the Twitter landscape. The scientific community is generally still a highly trusted source of information [63]. However, if disinformation and misinformation continue to be disseminated online, this could pose a legitimate threat to public health, as evidenced by the propaganda circulated during the 2014 Ebola outbreak [64] and 2020 coronavirus pandemic [65]. These realities require action, with a combination of regulation and health groups contributing to peer reviewed evidence and working with social media platforms to recognise and abate health information and disinformation. Offline, medical, and public health practitioners and researchers can work to dispel misinformation and disinformation directly through their built and trusted relationships and networks [63].

There are known and trusted strategies for addressing misinformation and disinformation in the field of health communication, but more research is needed to fully understand how well these translate into a social media context, how this information spreads online, and how to develop data-driven solutions to this growing threat [62,63,66]. It is important to assess the extent of misinformation and disinformation related to vaping, considering its potential to generate negative public health consequences. Deployment of innovative methods on a broader scale is needed, including natural language processing, assisted data mining, social network analysis, and online experimentation to track the spread of this content [62]. Surveillance endeavors must be agile and adaptable and require both researchers and practitioners to establish relationships with computer science professionals to stay abreast of the rapidly changing technology.

### Limitations

Coding using the triaxial classification system relied on the researchers’ subjective assessment, although the investigation of each tweet and user profile was particularly thorough and included examination of associated commentary to facilitate the understanding of the tweet context and examination of the user’s profile page including profile photo, bio, and recent activity. TrISMA’s programmed bot filtering processes were relied upon to remove data posted by questionable accounts. However, through our manual investigation some Twitter users were signposted as “suspected bot” accounts. Bot accounts have become more sophisticated over time, better aligning with human activity on Twitter [67], and as such, it was particularly difficult in some instances to ascertain whether some accounts were genuine users or not.

## Appendix

Multimedia Appendix 1 Coding framework: user category.

Multimedia Appendix 2 Coding framework: sentiment.

Multimedia Appendix 3 Coding framework: themes.

Multimedia Appendix 4 Results: all themes.

## Abbreviations

TrISMA: Tracking Infrastructure for Social Media Analysis
