# Supplementary material for: E-Cigarette Advocates on Twitter: Content Analysis of Vaping-Related Tweets
Source: JMIR Public Health Surveill. 2020 Oct 14;6(4):e17543. doi: 10.2196/17543 (PMC7593865; doi:10.2196/17543)
Supplement: Multimedia Appendix 1 [file publichealth_v6i4e17543_app1.docx]

Coding framework: user category.

| **User category** | **Definition** |
| --- | --- |
| Consumer advocacy group | A group whose main aim is to effect significant social change through the means of mobilisation. |
| E-cigarette advocate | Group or person whose timelines primary content is related to e-cigarettes. To further distinguish between e-cigarette advocates and the general public, these users may be identified by their admission of being a vaper, or vape or THR (tobacco harm reduction) advocate in their Twitter bio. |
| General public | Twitter account with a reasonable amount of posts and followers, and following a reasonable amount of people, (i.e. ratio of followers to followees). Their timeline spans a variety of topics that are not primarily e-cigarette related.[22] Twitter profile information and tweets reflect their individual thoughts and interests. |
| Government or politician | Government or political figures, groups or organisations. |
| Health or scientific group | Reputable health and scientific groups or organisations. |
| Medical doctor, nurse or group | A person, group or organisation who identifies as a physician, medical practitioner, medical doctor or nurse. |
| News and media sources | Person, group or organisation that focus on delivering news to the public. |
| Public health professional, researcher or academic | A person who identifies as a public health professional (besides medical doctor and nurse), researcher or academic. |
| Vape retailer or  manufacturer | Outlets and companies that sell or manufacturer e-cigarettes (online or physical store). |
| Suspected bot | “Accounts that appear to be fake or computerised, that are primarily promoting e-cigarette products (or other products); most accounts are disguised to appear as an ‘everyday person’.”[22]^(p3)^ To further “distinguish between human users and social bots, certain criteria such as information diffusion patterns (based on retweets or mentions), friend features (for example, the ratio of followers to followees), and content (frequency of nouns/verbs/adverbs in a tweet)”[44]^(p2)^ are used. |
| Account not active or user suspended | Account has been removed or suspended from Twitter. |
| Other | Any account that does not fit into the categories listed. |
