# Supplementary material for: E-Cigarette Advocates on Twitter: Content Analysis of Vaping-Related Tweets
Source: JMIR Public Health Surveill. 2020 Oct 14;6(4):e17543. doi: 10.2196/17543 (PMC7593865; doi:10.2196/17543)
Supplement: Multimedia Appendix 2 [file publichealth_v6i4e17543_app2.docx]

Coding framework: sentiment.

| **Sentiment** | **Definition** |
| --- | --- |
| Positive | Tweet is in favour of e-cigarettes and vaping, related products, and use. |
| Neutral | Tweet is not strong in either direction for or against e-cigarettes and vaping, related products, and use. |
| Negative | Tweet is against e-cigarettes and vaping, related products, and use. |
