# Supplementary material for: E-Cigarette Advocates on Twitter: Content Analysis of Vaping-Related Tweets
Source: JMIR Public Health Surveill. 2020 Oct 14;6(4):e17543. doi: 10.2196/17543 (PMC7593865; doi:10.2196/17543)
Supplement: Multimedia Appendix 3 [file publichealth_v6i4e17543_app3.docx]

Coding framework: themes.

| **Theme** | **Definition** |
| --- | --- |
| Addiction | E-cigarettes can be used to break nicotine or cigarette addiction, or reference to continued addiction to nicotine through e-cigarettes. |
| Advertising or promotion | Advertising, promotion and endorsement of vape products, brands, retailers, groups or events. Does not specifically need to be selling products to purchase, can include user-generated content of products they are using. |
| Airport regulation or policy | Reference to, or questions about airport regulation/policy relating to e-cigarettes. |
| Association with coffee or tea | Mentions or depicts coffee or tea with vaping. |
| Association with drugs | E-cigarettes mentioned or depicted in association with addictive or illicit substances other than nicotine. |
| Australian regulation or policy | Reference to, or questions about Australian regulation/policy relating to e-cigarettes. |
| Big pharma | Reference to the pharmaceutical industry’s involvement in e-cigarette regulation, and e-cigarettes compared to pharmacy cessation products. |
| Brand name | Mentions or depicts an e-cigarette brand name. |
| Celebrity use | Mentions or depicts a celebrity using or endorsing e-cigarettes. |
| Cessation or alternative – negative | Suggests e-cigarettes are not a tool to quit smoking cigarettes or other tobacco products; and should not be used as a substitution for tobacco products. |
| Cessation or alternative – neutral | Reference to e-cigarettes as a quit-smoking tool or substitution, however, is not strong in either direction, for or against. |
| Cessation or alternative – positive | Suggests e-cigarettes are a tool to quit smoking cigarettes or other tobacco products; and are a substitution for tobacco products. |
| Challenging current regulation or policy | Challenging or providing information to refute proposed or implemented e-cigarette regulation or policy. |
| Challenging anti-vaping commentary | Challenging or providing information contrary to negative reporting of e-cigarettes. Includes trolling behaviour. |
| Cost and savings compared to tobacco | Cost-saving or low cost of e-cigarettes compared to tobacco products. |
| Community or subculture | Conveys a vaping social identity, shared community affiliation or subculture. |
| Conspiracy against vaping | Suggests e-cigarettes are not being legalised due to protected tobacco or pharmaceutical trade; or conspired action by government and public health officials. |
| Craving | Expressing a craving, desire or need to use e-cigarettes. |
| Customer and retailer interaction | Customers providing feedback or engaging with retailers, and retailers directly engaging with customers. |
| Difference between  e-cigarettes and tobacco | Emphasising the difference between e-cigarettes and tobacco, and as such should not be regulated as tobacco products. |
| Doctor or nurse endorse or denounce | Medical doctors or nurses endorsing or denouncing e-cigarettes. |
| E-cigarette cessation | Ceasing e-cigarette use. |
| E-cigarette use or intent | Mentions or depicts someone using an e-cigarette, or their future intent to do so. |
| Effect on public health | Net public health gains or losses as a result of e-cigarettes. |
| E-liquid components | Mentions or depicts the components or ingredients in e-liquid. |
| Social promotion and recruitment | Attempts to engage other Twitter users in e-cigarette discussions, or to retweet content. |
| United States Food and Drug Administration (FDA) | Any mention of the US Food and Drug Administration. |
| Flavour | Mention or depiction of e-cigarette flavours. |
| Misc. e-cigarette information | Miscellaneous information about e-cigarettes. |
| Getting others started | Offers information on how to start using e-cigarettes or what products to use for beginners. |
| Giving or asking for advice | Offering or asking for advice about e-cigarettes. |
| Health and safety – negative | Negative health and safety aspects associated with vaping. |
| Health and safety – neutral | Health and safety aspects associated with vaping, however, is not strong in either direction, positive or negative. |
| Health and safety – positive | Positive health and safety aspects associated with vaping. |
| Hobby and DIY | Vaping as a hobby, building devices or coils, preparing e-liquids. |
| Humour and sarcasm | The use of humour or sarcasm concerning vaping or its users. |
| Indoor use | Mentions or depicts someone vaping indoors. |
| Issue with e-cigarettes | Mention of any issues or problems encountered using e-cigarettes (e.g. batteries, device malfunctions, e-liquid safety). |
| Marketing tactics | Mentioning methods used to promote and market e-cigarettes. |
| Nicotine | Any mention or depiction of nicotine. |
| Ordering product | A person has placed or received an e-cigarette order, or mentions shipping/delivery costs. |
| Pleasure | Pleasurable vaping experiences. |
| Price promotion | Coupons, percent off, discount offers, multi-buys and give-aways. |
| Pro advocacy | Use of, or describing vigorous campaigning to bring about political or social change in regards to e-cigarettes. |
| Product review | Consumers providing reviews of products, or retailers asking for consumers to review products. |
| Quality | Positive and negative comments about e-cigarette or associated products quality. |
| Regulation or policy – cautious | Expresses support for, or discusses cautious or restrictive e-cigarette regulation or policy. |
| Regulation or policy – liberal | Expresses support for, or discusses legalisation or liberal e-cigarette regulation or policy. |
| Regulation or policy – neutral | Mentions e-cigarette regulation/policy, however, is not strong in either direction, for or against. |
| Retailer name | Mentions or depicts an e-cigarette retailer by name. |
| Second-hand vape | Reference to second-hand smoke or vapour. |
| Sexualisation | Pairing e-cigarettes with sexually suggestive imagery or text. |
| Smoke-free | Mention that e-cigarettes are smoke-free or can be used to contravene smoke-free legislation. |
| Social capital | Explicit or implicit statement of increased or decreased social capital when using/since using e-cigarettes. |
| Statistics | Any mention of statistics concerning e-cigarettes. |
| Stigma and dislike e-cigarette user | Expression of dislike for e-cigarettes and their users, or the stigma/dislike some users have experienced. |
| Tax | Reference to proposed or implemented e-cigarette taxes, and how these taxes are used. |
| Australian Therapeutic Goods Administration (TGA) | Any mention of Australia’s Therapeutic Goods Administration. |
| Throat hit | Reference to throat hit - the sensation in the throat caused by nicotine as it's inhaled. |
| Tobacco or e-cigarette industry | Reference to the tobacco or e-cigarette industry. |
| Tobacco or e-cigarette initiation | Reference to never smokers or current smokers initiating e-cigarettes or e-cigarettes being a gateway to tobacco use. |
| Regulation or policy update | Reference to proposed or implemented e-cigarette regulation or policy. |
| Use in other populations | E-cigarette use among other populations (e.g. Indigenous, incarcerated). |
| Vape event | Reference to any event discussing or promoting e-cigarettes (e.g. scientific conference or a vaping convention). |
| Vape lounge | Space/shop where you can try e-cigarette products. |
| Vape play | Mentions or depicts vape tricks or cloud-chasing. Vape tricks are activities vapers undertake such as blowing smoke rings. Cloud-chasing is the activity of blowing large clouds of vapour using an e-cigarette. |
| Youth use | Reference to underage or youth using e-cigarettes. |
