# Supplementary material for: E-Cigarette Advocates on Twitter: Content Analysis of Vaping-Related Tweets
Source: JMIR Public Health Surveill. 2020 Oct 14;6(4):e17543. doi: 10.2196/17543 (PMC7593865; doi:10.2196/17543)
Supplement: Multimedia Appendix 4 [file publichealth_v6i4e17543_app4.docx]

Results: all themes.

| **Tweet content** | **2012 n (%)** | **2014 n (%)** | **2016 n (%)** | **2018 n (%)** | **Total n (%)** | **P value** |
| --- | --- | --- | --- | --- | --- | --- |
| Advertising or promotion | 268 (47.0) | 685 (57.3) | 633 (46.0) | 436 (33.8) | 2040 (46.0) | *P* < .001 |
| Price promotion | 77 (28.7) | 80 (11.7) | 152 (24.0) | 88 (20.2) | 397 (19.5) | *P* < .001 |
| Brand name | 124 (21.8) | 302 (25.3) | 448 (32.5) | 364 (28.2) | 1238 (27.9) | *P* < .001 |
| E-cigarette use or intent | 76 (13.3) | 254 (21.2) | 358 (26.0) | 282 (21.9) | 970 (21.9) | *P* < .001 |
| Cessation or alternative | 105 (18.4) | 182 (15.2) | 136 (9.9) | 293 (22.7) | 716 (16.2) | *P* < .001 |
| Positive | 100 (95.2) | 176 (96.7) | 130 (95.6) | 274 (93.5) | 680 (95.0) | *P* < .001 |
| Negative | 1 (1.0) | 4 (2.2) | 2 (1.5) | 13 (4.5) | 20 (2.8) | *P* = .004 |
| Neutral | 4 (3.8) | 2 (1.1) | 4 (2.9) | 6 (2.0) | 16 (2.2) | *P* = .270 |
| Health and safety | 67 (11.8) | 161 (13.5) | 139 (10.1) | 314 (24.4) | 681 (15.4) | *P* < .001 |
| Positive | 51 (76.1) | 114 (70.8) | 91 (65.5) | 198 (63.0) | 454 (66.7) | *P* < .001 |
| Negative | 10 (14.9) | 36 (22.4) | 36 (25.9) | 101 (32.2) | 183 (26.9) | *P* < .001 |
| Neutral | 6 (9.0) | 11 (6.8) | 12 (8.6) | 15 (4.8) | 44 (6.4) | *P* = .879 |
| Retailer name | 78 (13.7) | 234 (19.6) | 136 (9.9) | 201 (15.6) | 649 (14.6) | *P* < .001 |
| Flavour | 39 (6.8) | 145 (12.1) | 139 (10.1) | 184 (14.3) | 507 (11.4) | *P* < .001 |
| Views on regulation or policy | 6 (1.1) | 45 (3.8) | 64 (4.6) | 192 (14.9) | 307 (6.9) | *P* < .001 |
| Liberal | 3 (50.0) | 36 (80.0) | 58 (90.6) | 151 (78.6) | 248 (80.8) | *P* < .001 |
| Cautious | 3 (50.0) | 6 (13.3) | 5 (7.8) | 40 (20.8) | 54 (17.6) | *P* < .001 |
| Neutral | 0 (0) | 3 (6.7) | 1 (1.6) | 1 (0.5) | 5 (1.6) | *P* = .562 |
| Community or subculture | 18 (3.2) | 48 (4.0) | 84 (6.1) | 155 (12.0) | 305 (6.9) | *P* < .001 |
| Nicotine | 19 (3.3) | 42 (3.5) | 89 (6.5) | 143 (11.1) | 293 (6.6) | *P* < .001 |
| Social promotion and recruitment | 30 (5.3) | 57 (4.8) | 100 (7.3) | 103 (8.0) | 290 (6.5) | *P* = .004 |
| Challenging current regulation or policy | 6 (1.1) | 41 (3.4) | 97 (7.0) | 119 (9.2) | 263 (5.9) | *P* < .001 |
| Product review | 25 (4.4) | 57 (4.8) | 107 (7.8) | 68 (5.3) | 257 (5.8) | *P* = .002 |
| Regulation or policy update | 12 (2.1) | 72 (6.0) | 62 (4.5) | 99 (7.7) | 245 (5.5) | *P* < .001 |
| Australian regulation or policy | 5 (0.9) | 41 (3.4) | 65 (4.7) | 125 (9.7) | 236 (5.3) | *P* < .001 |
| Pro advocacy | 4 (0.7) | 31 (2.6) | 60 (4.4) | 95 (7.4) | 190 (4.3) | *P* < .001 |
| Humour and sarcasm | 8 (1.4) | 36 (3.0) | 80 (5.8) | 64 (5.0) | 188 (4.2) | *P* < .001 |
| Getting others started | 55 (9.6) | 49 (4.1) | 35 (2.5) | 36 (2.8) | 175 (3.9) | *P* < .001 |
| Tobacco or e-cigarette industry | 23 (4.0) | 45 (3.8) | 29 (2.1) | 75 (5.8) | 172 (3.9) | *P* < .001 |
| Conspiracy against  vaping | 2 (0.4) | 28 (2.3) | 64 (4.6) | 57 (4.4) | 151 (3.4) | *P* < .001 |
| Youth use | 3 (0.5) | 22 (1.8) | 25 (1.8) | 92 (7.1) | 142 (3.2) | *P* < .001 |
| Ordering product | 33 (0.7) | 37 (0.8) | 25 (0.6) | 33 (0.7) | 128 (2.9) | *P* < .001 |
| Tobacco or e-cigarette initiation | 8 (1.4) | 29 (2.4) | 19 (1.4) | 68 (5.3) | 124 (2.8) | *P* < .001 |
| Hobby and DIY | 14 (2.5) | 41 (3.4) | 30 (2.2) | 36 (2.8) | 121 (2.7) | *P* = .267 |
| Effect on public health | 3 (0.5) | 10 (0.8) | 24 (1.7) | 84 (6.5) | 121 (2.7) | *P* < .001 |
| Giving and asking for advice | 27 (4.7) | 14 (1.4) | 34 (2.5) | 32 (2.5) | 110 (2.5) | *P* = .001 |
| Issue with e-cigarettes (i.e. malfunction, e-liquid safety) | 6 (1.1) | 32 (2.7) | 31 (2.3) | 38 (2.9) | 107 (2.4) | *P* = .088 |
| Association with drugs | 16 (0.4) | 12 (0.3) | 28 (0.6) | 47 (1.1) | 103 (2.3) | *P* < .001 |
| Quality | 14 (2.5) | 44 (3.7) | 27 (2.0) | 13 (1.0) | 98 (2.2) | *P* < .001 |
| Indoor use | 14 (2.5) | 30 (2.5) | 13 (0.9) | 39 (3.0) | 96 (2.2) | *P* = .002 |
| Challenging anti-vaping commentary | 0 (0) | 25 (2.1) | 34 (2.5) | 29 (2.2) | 88 (2.0) | *P* = .003 |
| E-liquid components | 0 (0) | 17 (1.4) | 36 (2.6) | 34 (2.6) | 87 (2.0) | *P* < .001 |
| Addiction | 11 (1.9) | 5 (0.4) | 13 (0.9) | 53 (4.1) | 82 (1.9) | *P* < .001 |
| United States Food and Drug Administration (FDA) | 0 (0) | 7 (0.6) | 33 (2.4) | 42 (3.3) | 82 (1.9) | *P* < .001 |
| Stigma and dislike of e-cigarette users | 4 (0.7) | 17 (1.4) | 33 (2.4) | 27 (2.1) | 81 (1.8) | *P* = .044 |
| Misc. e-cigarette information | 17 (3.0) | 30 (2.5) | 16 (1.2) | 15 (1.2) | 78 (1.8) | *P* = .003 |
| Marketing tactics | 6 (1.1) | 14 (1.2) | 23 (1.7) | 32 (2.5) | 75 (1.7) | *P* = .042 |
| Difference between and e-cigarettes and tobacco | 3 (0.5) | 12 (1.0) | 29 (2.1) | 30 (2.3) | 74 (1.7) | *P* = .005 |
| Customer and retailer interaction | 16 (2.8) | 18 (1.5) | 14 (1.0) | 19 (1.5) | 67 (1.5) | *P* = .034 |
| Vape play | 2 (0.4) | 21 (1.8) | 22 (1.6) | 21 (1.6) | 66 (1.5) | *P* = .117 |
| Pleasure | 11 (1.9) | 20 (1.7) | 14 (1.0) | 17 (1.3) | 62 (1.4) | *P* = .343 |
| Celebrity use | 7 (1.2) | 10 (0.8) | 14 (1.0) | 24 (1.9) | 55 (1.2) | *P* = .101 |
| Social capital | 6 (1.1) | 17 (1.4) | 12 (0.9) | 7 (0.5) | 42 (0.9) | *P* = .154 |
| Cost and savings compared to tobacco | 13 (2.3) | 7 (0.6) | 1 (0.1) | 19 (1.3) | 40 (0.9) | *P* < .001 |
| Doctor or nurse endorse or denounce | 1 (0.2) | 4 (0.3) | 12 (0.9) | 24 (1.9) | 41 (0.9) | *P* < .001 |
| Second-hand vape | 9 (1.6) | 5 (0.4) | 14 (1.0) | 12 (0.9) | 40 (0.9) | *P* = .099 |
| Vape event (i.e. vape convention, academic conference) | 5 (0.9) | 7 (0.6) | 17 (1.2) | 9 (0.7) | 38 (0.9) | *P* = .292 |
| Tax | 2 (0.4) | 3 (0.3) | 9 (0.7) | 18 (1.4) | 32 (0.7) | *P* = .004 |
| Statistics | 0 (0) | 4 (0.3) | 2 (0.1) | 23 (1.8) | 30 (0.7) | *P* < .001 |
| Use in other populations | 0 (0) | 0 (0) | 6 (0.4) | 21 (1.6) | 27 (0.6) | *P* < .001 |
| Smoke-free | 15 (2.6) | 5 (0.4) | 3 (0.2) | 3 (0.2) | 26 (0.6) | *P* < .001 |
| Sexualisation | 3 (0.5) | 10 (0.8) | 11 (0.8) | 1 (0.1) | 25 (0.6) | *P* = .039 |
| Big pharma | 0 (0) | 7 (0.6) | 4 (0.3) | 13 (1.0) | 24 (0.5) | *P* = .019 |
| Craving | 6 (1.1) | 5 (0.4) | 9 (0.7) | 3 (0.2) | 23 (0.5) | *P* = .116 |
| Vape lounge | 2 (0.4) | 7 (0.6) | 3 (0.2) | 3 (0.2) | 15 (0.3) | *P* = .376 |
| Airport regulation or policy | 0 (0) | 4 (0.3) | 8 (0.6) | 2 (0.2) | 14 (0.3) | *P* = .142 |
| Association with coffee or tea | 1 (0) | 1 (0) | 7 (0.2) | 4 (0.1) | 13 (0.3) | *P* = .263 |
| E-cigarette cessation | 0 (0) | 2 (0.2) | 2 (0.1) | 3 (0.2) | 7 (0.2) | *P* = .876 |
| Throat hit | 0 (0) | 0 (0) | 2 (0.1) | 4 (0.3) | 6 (0.1) | *P* = .208 |
| Australian Therapeutic Goods Administration (TGA) | 1 (0.2) | 0 (0) | 1 (0.1) | 1 (0.1) | 3 (0.1) | *P* = .612 |
